# Pioneering contribution of Professor Bruce Ames to early development in biochemical aspects of oxidatively generated damage to DNA

**DOI:** 10.3389/fmolb.2025.1636255

**Published:** 2025-08-20

**Authors:** Jean Cadet, J. Richard Wagner

**Affiliations:** Department of Medical Imaging and Radiation Sciences, Faculty of Medicine and Health Sciences, Université de Sherbrooke, Sherbrooke, QC, Canada

**Keywords:** oxidatively generated damage to DNA, reactive oxygen and nitrogen species, hydroxyl radical, one-electron oxidants, singlet oxygen, cellular DNA oxidation, measurement oxidized nucleosides, urinary DNA oxidation lesions

## Abstract

The first part of the memorial review article is devoted to a retrospective of selected topics that were the subject of pioneering studies over the period 1985–2025 by Professor Bruce Ames. Major efforts were made to develop accurate and sensitive assays including HPLC coupled with electrochemical detection for monitoring the formation of 8-oxo-7,8-dihydroguanine in isolated cells and animal tissues. Special attention was provided to the minimization of artefactual oxidation of DNA that occurs during sample preparation. Complementary information on the biological relevance of 8-oxo-7,8-dihydroguanine and 5,6-dihydroxy-5,6-dihydrothymine was gained from the non-invasive measurement of the oxidized bases and nucleosides in various mammalian fluids. The second part of this review focuses on the current situation concerning the formation of oxidized bases in cellular DNA produced under various conditions of oxidative stress and enzymatic ten-eleven TET-oxidation of 5-methylcytosine. The analysis of DNA base modifications by LC-MS/MS is the gold standard for the quantitative monitoring of base oxidation products in both DNA and several body fluids; oxidizing conditions that may not be suitable for biological studies. Low levels of oxidatively-induced lesions in cells are difficult to assess by chromatographic and MS methods because of a significant increase in the yields of oxidized bases/nucleosides above the background level including a significant contribution of adventitious oxidation reactions that cannot be totally suppressed. In a complementary way, the application of modified versions of the comet assay and alkaline elution techniques that target general classes of DNA lesions provides a more global account of damage although it gives less structural information about DNA damage formed under chronic exposure to mild oxidizing conditions.

## 1 Introduction

Professor Bruce Ames made a major seminal discovery with the report more than 50 years ago of the *Salmonella* bacterial/microsome ‘Ames test’ (Ames et al., 1973; McCann et al., 1975), which is a widely applied assay for assessing the genotoxicity and mutagenicity of chemicals (Marnett, 2019; Rueff et al., 2019; Lipsick, 2021; Large et al., 2023). During his career and life, Pr. Ames also made several other significant contributions in related domains of research. These include chemical carcinogenesis, free radical theories of aging, mechanisms of protection against oxidative stress, and the role and function of dietary micronutrients (Smith et al., 2021; Ames, 2022). This review article is aimed at highlighting the pioneering studies of Pr. Bruce Ames and his associates into the chemical and biochemical aspects of oxidatively generated damage to DNA. In the second part of the article the current situation on the formation of oxidatively generated damage in cellular DNA and the release of oxidized nucleobases/nucleosides in biological fluids (urine, plasma) as non-invasively measured biomarkers of oxidative stress is critically reviewed. This includes a survey of the capabilities and limits of available chromatographic and enzymatic based methods for the accurate measurement of DNA oxidation modifications in biological samples.

## 2 Major contributions of Dr. Ames’s laboratory

The laboratory of Bruce Ames (the Ames lab) was an exciting place to do research during post-doctoral studies (1989–1992). The lab included about 6-8 post-docs, 1-2 graduate students and technical assistants. Everyone was entitled to 3 feet of lab counter and 3 feet of office space squeezed in between instruments, and a common room with computers for those in the stage of writing up their results. The lab would operate 24 h a day, and if you checked in late at night, you could see the lights flashing from the instruments working through overnight analyses. Every week, we would gather around a conference table on the fourth floor of Barker Hall with our lunch, which was often with a slice of pizza from Shattuck Av., and present our results for timely analysis and discussion. The topics of the day were very diverse and included, among others: ascorbate inhibition of lipid peroxidation, *in vitro* mutagenesis in *Salmonella typhimurium*, urinary excretion of DNA repair products, and others. When Bruce was away, he would call constantly to check in with everyone and bring him up to date about the latest results, anxious to hear about our most recent discoveries. At this time, the lab devoted enormous effort to develop assays toward the molecular analysis of oxidatively induced damage in biological contexts, particularly damage associated with DNA, which constituted a potential source of mutations in humans.

### 2.1 Analysis of DNA damage

#### 2.1.1 8-oxo-7,8-dihydro-2′-deoxyguanosine (8-oxodG)

Early studies in the 1980s–90s used GC-MS to measure individual DNA base damage, which involved the hydrolysis of DNA using strong acid and heat followed by derivatization of the released nucleobases and GC-MS analyses (Dizdaroglu, 1985). During this time, however, many groups including the Ames lab turned to a novel assay for 8-oxodG using HPLC coupled to an electrochemical detector (HPLC-ECD) (Floyd et al., 1988). The inclusion of enzymatic digestion in these analyses circumvented the necessity to expose DNA to harsh acid to break it down into its component monomers. Furthermore, nucleosides and their modifications are well separated by reversed phase HPLC in contrast to nucleobase derivatives. Using this approach, the Ames lab reported several interesting findings: they reported that 8-oxodG increased by 1.5-3-fold in the DNA of rat tissues with age from 2 to 24 months old (Fraga et al., 1990). They also reported the ability of ascorbate to decrease the level of 8-oxodG in human sperm DNA (Fraga et al., 1991) and marked increases of 8-oxodG in transgenic mice with chronic active hepatitis (Hagen et al., 1994). However, there was unappealing concern about the contribution of artifactual oxidation in the analysis of 8-oxodG in biological samples. The conclusions of a multicenter study in which the steady state level of 8-oxodG was measured in mammalian tissue showed enormous variations by as much as three orders of magnitude depending on the method and laboratory of analysis (Collins et al., 2004; Gedik et al., 2005). The Ames group devoted much time and effort to optimize the method of DNA extraction and subsequent steps in preparation for HPLC-ECD analysis (Helbock et al., 1998; Helbock et al., 1999). Using this method, Helbock et al. reported steady state levels of 8-oxodG in the DNA of rat liver that were more consistent and gave considerably lower values by about 10-fold compared to those previously published. Chaotropic salts, such as NaI, disrupt the hydration shell of DNA and help remove contaminants, such as potentially damaging metal ions. The use of NaI to precipitate DNA was later proven to be one of the most effective methods to reduce artefactual oxidation of DNA during its extraction from cells (Ravanat et al., 2002; and references in Section 3). The apparent fold increases of 8-oxodG in rodent tissues with age reported by the Ames group were later confirmed by using NaI in combination with HPLC and tandem MS (Gan et al., 2012). An alternative method was later proposed by Beckman and Ames in which the base moiety of 8-oxodG was selectively removed from DNA by treatment with *E. coli* repair enzyme formamidopyrimidine (Fapy) DNA glycosylase (Beckman et al., 2000). The modified base was then quantified by HPLC-EC giving steady state values of 0.4 8-oxodG/10^6^ dG for the DNA of Hela cells in culture. Although a comparison with previous method with complete digestion was not carried out, the selective digestion with Fpg considerable reduced the observed level of damage. An advantage of the latter method is that it minimizes the autooxidation of dG to 8-oxodG and permits the injection of relatively high equivalent amounts of DNA. Although the problem of artifactual oxidation still exist today, the gold standard for the analysis of specific modifications of DNA has become HPLC or UPLC coupled to tandem mass spectrometry (LC-MS/MS) for the analysis of 8-oxodG and other potential markers of DNA damage.

#### 2.1.2 Uracil

Much effort in the Ames lab was invested toward the analysis of uracil (Ura) in DNA from biological samples. The basic hypothesis was that a deficiency in folate and/or Vitamin B6 induces DNA strand breaks due to an inhibition of thymidylate synthesis and the incorporation of uracil instead of thymine into DNA. It should also be noted that Ura is also formed by deamination of the one-electron oxidation mediated radical cation of cytosine as shown in model studies (Decarroz et al., 1987). During the 90s, the Ames group developed a novel assay for the analysis of Ura in DNA using uracil DNA glycosylase (UDG) to selectively excise Ura from DNA, followed by derivatization of Ura with 3,5-bis(trifluoromethyl)benzyl bromide and its analysis by GC-MS (Blount and Ames, 1994). They used this assay to show that Ura levels in the DNA of RBCs together with micronuclei frequencies were elevated in folate deficient subjects and reduced in folate supplementation (Blount et al., 1997). The initial assay was improved upon giving a limit of detection in the low fmol range and the ability to detect Ura in as little as 5 ug of lymphocyte DNA (Mashiyama et al., 2004; Mashiyama et al., 2008). With the new assay, this group studied changes of Ura in DNA with folate status upon supplementation of lymphocytes in culture with folate and/or nucleosides. More recent studies, using LC-MS/MS with isotopic dilution, report levels of 0.15 Ura and 0.08 Ura/10^6^ N in the DNA of mouse embryonic fibroblasts and human lymphoblastoid cell lines, respectively (Galashevskaya et al., 2013). The widely different concentrations of reported Ura in DNA may be due to artefacts during sample preparation and analyses, such as the presence of trace amounts of cytidine deaminase activities in biological samples. Using single molecule sequencing, the level of Ura in murine and human genomes appears to be small even lower than recent LC-MS/MS analysis. (Liu et al., 2024). These results suggest that the activity of uracil glycosylases is very efficient for intact cells *in vivo*. It is not known whether the same conclusion may be made for oxidatively-induced DNA damage.

### 2.2 Analysis of DNA repair products in urine

Early studies in the 1990s provided estimates of thymine glycol in urine based on a method that converted thymine glycol into thymine (Cathcart et al., 1984). The method involved initial purification of thymine and thymidine glycols by phenylboronate affinity chromatography, conversion of saturated 5,6-glycols to thymine using hydroiodic acid, followed by reversed phase HPLC with UV detection. Using this method, the Ames group reported a correlation between oxygen consumption and the excretion of thymine and thymidine glycols such that mice excreted 4.5-fold more than monkeys, which excreted 4-fold more than humans (Adelman et al., 1988; Ames, 1989). With the availability of a new sensitive detection method for 8-oxoGua derivatives (HPLC-EC), the Ames group turned to the analysis of this modification in urine samples. To help in the analysis of 8-oxoGua in biological fluids, such as urine, much time and effort was devoted to the development and characterization of antibodies toward 8-oxoGua (Park et al., 1992; Degan et al., 1991; Shigenaga et al., 1994). Using a monoclonal antibody and HPLC-EC to separate 8-oxo-7,8-dihydroguanine derivatives, the Ames group estimated the levels of 8-oxoGua, 8-oxo-7,8-dihydroguanosine (8-oxorG) and 8-oxodG in various biological fluids, including spent medium from culture, plasma, and urine. Thereby, the levels of urinary 8-oxoGua, 8-oxorG and 8-oxodG were estimated to be 4,000, 910, and 410 pmol/kg/day, respectively, for rats on a nucleic acid free diet. The combined level of urinary 8-oxoG and 8-oxodG (4,300 pmol/kg/day), which was considered to represent the excision of 8-oxo-7,8-dihydroguanine modifications from DNA, suggests that rat cells on the average are subjected to about 100,000 oxidative hits to DNA per day (this estimate assumes that 8-oxoxdG represents 5% of the total oxidatively-induced lesions to DNA) (Fraga et al., 1990; Shigenaga and Ames, 1991). Although the monoclonal antibody (Fab 166) was rapid and efficient for the isolation of 8-oxoGua derivatives from urine, the antibody exhibited relatively high cross-reactivity with a number of other compounds with a similar structure including uric acid and related adenine derivatives. Building on this approach, the Ames group in collaboration with others designed antibody conjugates that recognize DNA containing 8-oxoGua in real time imaging using confocal scanning laser microscopy; the authors showed that reported increases of immunoreactivity of 8-oxodG in control cultures and increases within the nucleus and mitochondria of cells in culture treated with H_2_O_2_ or radiation (Soultanakis et al., 2000). The early achievements of Pr. Ames and his collaborators in the highly competitive domain of developing methods to measure oxidatively-induced damage to DNA and relate the levels to physiological outcomes significantly impacted and facilitated the advancement of future research in this area.

## 3 Current status on oxidatively generated damage in cellular DNA and biological fluids

The analysis of DNA damage *in vivo* was and still is a topic of major interest as illustrated by several recent developments in the measurement, formation and repair of oxidatively damage to cellular DNA. This section deals with the current situation concerning the unambiguous identification and accurate measurement of base oxidation lesions in cellular DNA upon exposure to hydroxyl radical (^**.**^OH), one-electron oxidants and singlet oxygen (^1^O_2_). UVA/Visible light and ionizing radiations constitute two major sources of external oxidants (O’Neill and Wardman, 2009; Cadet et al., 2017; Di Mascio et al., 2019), whereas oxygen metabolism is able under physiological conditions to continuously oxidize DNA via the initial release of superoxide anion radical (O_2_·-) mediated by mitochondrial respiration and subsequent conversion into hydrogen peroxide (Sies et al., 2022) a major cellular redox signaling molecule (Sies, 2021). The generation of O_2_·- and also of ·NO, a reactive nitrogen species (RNS), is exacerbated under several conditions of oxidative stress including inflammation and phagocytosis (Møller et al., 2014; Flint et al., 2016). In addition, ten eleven translocation (TET) dioxygenases oxidize 5-methylcytosine as part of an active demethylation pathway (Tahiliani et al., 2009; Kriaucionis and Heinz, 2009; DeNizio et al., 2021; Feng et al., 2021; Zhang et al., 2023). 5-Hydroxymethylcytosine (5-HmCyt), thereby generated, is subsequently converted by iterative oxidation into 5-formylcytosine (5-FoCyt) and 5-carboxylcytosine (5-CaCyt) (He et al., 2011; Ito et al., 2011); the latter products are subsequently removed from DNA by thymidine DNA glycosylase (TDG) a base excision repair (BER) protein before cytosine insertion (Maiti and Drohat, 2011; Kohli and Zhang, 2013; Zhongb and Sczepanski, 2023; Schnable et al., 2024). A direct decarboxylation reaction of 5-CaCyt has been recently proposed to occur in cells according to an incompletely understood mechanism (Feng et al., 2021). Recent progress on the measurement of released oxidized nucleobases and nucleosides in biological fluids, mostly urine and to a lesser extent plasma, are also reported as indirect and non-invasive approaches to estimate the occurrence of oxidatively generated damage to nucleic acids in humans and animals (Chao et al., 2021).

### 3.1 Measurement of nucleobase base oxidation lesions in cellular DNA and biological fluids

The measurement of oxidatively generated damage to cellular DNA remains a challenging issue and has been the subject of numerous studies during the last 50 years. Early attempts initially failed using chemical assays based on either the reduction or alkaline degradation of 5,6-dihydroxy-5,6-dihydrothymine (ThyGly) (Hariharan and Cerutti, 1972; Hariharan and Cerutti, 1977; Cadet and Berger, 1985; Cadet et al., 2011). Both methods required pre-labeling of cellular DNA with either [^3^H]- or [^14^C]-thymine, which led to auto-radiolysis and elevated artefactual formation of oxidized base modifications close to one lesion per 10^3^ nucleosides. Other early developed methods including GC-MS (Dizdaroglu et al., 1991), immunological assays (Yin et al., 1995; Murphy et al., 2022), [^32^P]-post-labeling techniques (Devanaboyina and Gupta, 1996) have been questioned for their lack of accuracy and/or occurrence of major drawbacks as discussed in comprehensive review articles (Cadet et al., 1997a; Cadet et al., 2004). Although the initial version of gas chromatography-mass spectrometry (GC-MS) method has recently benefited by a few improvements, the method is still insufficient and suffers from several major flaws (Cadet et al., 1997b). These include as the main questionable issues the degradation of several unstable oxidized bases during acid hydrolysis of extracted DNA from cells and significant artefactual oxidation of overwhelming canonical bases with an averaged 10^−4^ frequency of formation during the derivatization step prior to CG-MS analysis (Hamberg and Zhang, 1995; Ravanat et al., 1995; Douki et al., 1996). The latter drawback mostly explains the high discrepancy, by at least an order of magnitude (Halliwell and Dizdaroglu, 1992) between the yields of 8-oxoGua yields determined by GC-MS (Dizdaroglu, 1993) and the more accurate high-performance liquid chromatographic analytical method coupled with either electrochemical detection (HPLC-ECD) (Floyd et al., 1986) or high resolution tandem mass spectrometry with electrospray ionization detection (LC-MS/MS) (Cadet et al., 2002). These discrepiancies are discussed below.

#### 3.1.1 HPLC methods

The discovery of 8-oxo-7,8-dihydroguanine (8-oxoGua) as an oxidation product of guanine under exposure to Udenfriend reagent (Kasai and Nishimura, 1984) was followed by the development of the HPLC-ECD assay using an amperometric/coulometric detector in the one-oxidation mode (Floyd et al., 1986). Thus, the method that is able to detect other low oxidation potential modified bases including 8-oxo-7,8-dihydroadenine (8-oxoAde) (Berger et al., 1990), 2,6-diamino-4-hydroxy-5-formamidopyrimidine (FapyGua), 4,6-diamino-5-formamidopyrimidine (FapyAde) (Park et al., 1989), 5-hydroxycytosine (5-OHCyt) and 5-hydroxyuracil (5-OHUra) (Wagner et al., 1992) and related nucleosides was widely applied to measure 8-oxoGua and 8-oxo-7,8-dihydro-2′-deoxyguanosine (8-oxodG) in cellular DNA and biological samples. The HPLC-ECD method was gradually replaced at the beginning of 2000 by the advent of the versatile and accurate HPLC analytical tool coupled with electrospray ionization tandem mass spectrometry (LC-MS/MS) recognized as the gold standard method for monitoring the formation of base/nucleoside oxidation products in cellular DNA, RNA and biological fluids (Cadet and Poulsen, 2010; Tretyakova et al., 2013; Chao et al., 2021). The first applications of this analytical method to biological samples concerned the measurement of 8-oxodG in the DNA of rat (Serrano et al., 1996) and pig liver (Ravanat et al., 1998) as well as in urine. This was subsequently extended to the quantitative detection of several modified 2′-deoxyribonucleosides released from extracted DNA using optimized conditions of enzymatic hydrolysis and HPLC separation upon exposure of cellular DNA to ionizing radiation (Frelon et al., 2000). The modifications included the four *cis* and *trans* diastereomers of dTGly, 5-(hydroxymethyl)-2′-deoxyuridine (5-HmdU), 5-formyl-2′-deoxyuridine (5-FodU), 8-oxo-7,8-dihydro-2′-deoxyadenosine (8-oxodA). A slightly different protocol was used for the measurement of 2,6-diamino-4-hydroxy-5-formamidopyrimidine (FapyGua) and 4,6-diamino-5-formamidopyrimidine (FapyAde) that arise from the quantitative hydrolysis of unstable released 2′-deoxyribonucleoside precursors from enzymatically digested DNA (Douki et al., 1997). Multiple reaction monitoring (MRM) detection provides relevant structural insights about the lesions through characteristic fragmentation showing a pseudo-molecular ion and its daughter ions produced by collision-induced dissociation (CID). The MRM mode that can be used either in the positive or negative ESI fragmentation according to the analyzed lesions (Frelon et al., 2000) ensures also a reduction in the background signal and therefore an increase in the detection sensitivity (Cadet et al., 2002). Furthermore the use of [^13^C] and [^15^N]-labeled internal standards at the preference of deuterated compounds allows for *in situ* calibration of the measurements through isotopic dilution, which constitutes the current way to monitor the formation of a wide range of modified nucleobase/nucleoside lesions from chemically and enzymatically oxidized cellular DNA (Cadet and Wagner, 2013; Cadet et al., 2017).

The LC-MS/MS tool has progressively replaced HPLC-ECD and GC-MS assays (for an extensive review on early findings, see Cadet et al., 2017; Chao et al., 2021) for the detection of 8-oxoGua, related nucleosides, and thymine oxidation products in several fluid matrices including urine, plasma, saliva (Weimann et al., 2001; Cooke et al., 2009). This powerful analytical approach has been shown to be more accurate than the enzyme-linked immunosorbent assay (ELISA) for the detection of 8-oxodG in biological fluids (European Standards Committee on Urinary (DNA) Lesion Analysis, 2010). However, it has been shown that a monoclonal antibody recognizing both 8-oxodG and 8-oxo-7,8-dihydroguanosine (8-oxorG) can be utilized in a column for the pre-purification of complex whole urine samples (Park et al., 1992) before HPLC-ECD analysis (Degan et al., 1991). Several improved strategies have been used to take care of the matrix complexity of the fluid samples that may lead to ion suppression and therefore a loss of signal in MS analysis. One approach that was initially applied to the measurement of 8-oxodG in cellular DNA (Chao et al., 2008) involves the use of on-line solid phase extraction (Hu et al., 2010). Furthermore, two-dimensional LC (2D) MS/MS instruments equipped with a switching valve allows for the transfer of target analytes from a trap column to a second column before MS detection. This method has been implemented for the detection of a wide range of excreted oxidized bases, mostly derived from guanine and 5-methylcytosine (Rozalski et al., 2016; Shih et al., 2018; Skalska-Bugala et al., 2022). Another mass screening strategy for the detection of 8-oxoGua and 8-oxodG involves triple quadrupole tandem mass spectrometry (LC-QqQ-MS/MS) that operates through ESI in selected reaction monitoring (SRM) mode by collision-induced dissociation (CNL) scanning (Cooke et al., 2018). High resolution mass spectrometry (HR-MS) detection has also been considered as an accurate mass measurement at the level of a few ppm. A hybrid quadrupole-linear ion trap-orbitrap mass spectrometry (Q-LIT-OT-MS) instrument using dd Nl-MS3 scan was recently applied for the high resolution measurement of systemic urinary modified nucleosides, including 8-oxodG and 8-oxodA in cellular DNA (Chang et al., 2021).

The accurate LC-MS/MS method in the MRM mode with isotope dilution shows a fair level of sensitivity, within the low femtomole range, for the detection of 8-oxodG and other oxidized 2′-deoxyribonucleosides in cellular DNA and urinary samples. However, a major limitation of chromatographic methods that involves DNA extraction and subsequent work-up concerns the occurrence of spurious oxidation reactions. Such oxidation is likely the result of Haber-Weiss/Fenton reactions that artifactually increase the level of baseline lesions and therefore questions the accuracy of the measurements. This observation was already made in the early 90s (Harris et al., 1994; Helbock et al., 1998; Helbock et al., 1999) and has been the subject of debate and various attempts to minimize/prevent adventitious oxidation of overwhelming canonical nucleobases/nucleosides. For this purpose, the NaI chaotropic DNA extraction method was used together with the addition of antioxidants, metal ion chelators and/or radical scavengers to aqueous solutions of enzymatic digests following DNA isolation (Ravanat et al., 2002; Ravanat et al., 2004; Chao et al., 2008; Mangal et al., 2009). Using these procedures with HPLC-ECD/LC-MS/MS usually give reduced steady-state levels of 8-oxodG; however, the levels are still several fold higher than the yields of formamidopyrimidine DNA-*N*-glycosylase (Fpg)-sensitive sites (8-oxoGua, FapyGua and 8-oxoAde) as monitored by either the alkaline elution technique or the alkaline comet assay. This was unambiguously established by a large inter-laboratory study involving the European Standard Committee on Oxidative DNA Damage (ESCODD, 2002; Collins et al., 2004; Gedik et al., 2005). It was shown that evaporation to dryness of the aqueous solution of digested DNA filtrate of pre-purified enzymatically digested DNA on a SPE column led to a significant increase in the level of 8-oxodG (Chao et al., 2008). Another critical factor concerns the amount of extracted DNA for HPLC analysis since an inverse correlation was noted between the yield of 8-oxodG measured by LC-MS/MS and the amount of DNA in sperm samples (Badouard et al., 2008). In this respect, it was recommended that the size of analyzed DNA samples should be higher than 50 µg in order to minimize the contribution of spurious oxidation as outlined in an earlier report (Beckman and Ames, 1996; Helbock et al., 1999).

Noteworthy is the occurrence of spurious oxidation to nucleobases and 2-deoxyribose components of DNA once extracted and then digested in aqueous solution, which cannot be totally prevented despite various optimization attempts. Consequently, the robust LC-MS/MS and HPLC-ECD methods are not able to accurately assess low chronic damaging effects of endogenous and exogenous oxidizing agents on cellular DNA. For example, HPLC-ECD was unable to detect significant increases in the level of 8-oxodG above the baseline level in cellular DNA unless cells were exposed to high doses of radiation of at least 60 Gy (Pouget et al., 1999). Interestingly, the detection threshold for a significant increase in the level of Fpg-sensitive sites is only 0.5 Gy allowing one to establish a linear formation of the purine lesions within the 0.5–10 Gy dose range (Sauvaigo et al., 2003). Similar observations were made for other main radiation-induced oxidized nucleosides including dTGly, 5-HmdU, 5-FodU, 8-oxodA, and several dC modified products since doses higher than 150 Gy are necessary for their unambiguous detection in cellular DNA.

This major limitation clearly indicates that HPLC-ECD and optimized LC-MS/MS are generally not appropriate for measuring small increases in the frequency of oxidatively generated base damage in cellular DNA because of interferring spurious oxidation during DNA extraction and subsequent work-up. The results thus far indicate that the use of HPLC based analytical tools as well as modified GC-MS methods involving an enzymatic digestion step may not be able to assess the damaging effects to cellular DNA of chronic exposure to environmental oxidizing agents and biological processes such as oxidative metabolism, phagocytosis, inflammation and cancer progression. In contrast, acute conditions of oxidative stress including high doses of UVA/ionizing radiations and elevated concentrations of oxidants can generate a significant elevation in the levels of DNA damage above the cellular steady-state background and contribution from artefactual oxidation. As long as dose responses are linear, the results may be extrapolated to low doses; however, it is necessary to establish responses of damage at low doses and take into account biological factors, such as DNA repair, which determine the steady state level.

#### 3.1.2 DNA repair glycosylase based assays

The characterization and gene cloning of several DNA-glycosylases (UNG, APE, Endo III, Fpg, OGG1, etc.) (Boiteux et al., 1987; Boiteux et al., 1990; van der Kemp et al., 1996; Radicella et al., 1997) that initiate the base excision repair pathway of purine and pyrimidine oxidized bases (Tchou et al., 1991; Wallace, 1994; David et al., 2007; Beard et al., 2019) has provided a strong impetus to the development of sensitive methods of detection of oxidatively generated damage in single cells. This was mostly achieved by using either the alkaline comet assay (Gedik et al., 1998; Collins et al., 2023) or the alkaline elution technique (Pflaum et al., 1997; Epe, 2012) for generating additional single strand breaks following DNA *N*-glycosylase-induced formation of abasic sites. Thus, bacterial Fpg is able to excise, in addition to FapyGua, 8-oxoGua and FapyAde, whereas human and yeast 8-oxoguanine glycosylase (OGG1) is more specific since it only recognizes oxidatively generated guanine modifications. In addition, endonuclease III (Endo III), which exhibits a wider range of substrate specificity than Fpg and OGG1, has been used to detect pyrimidine modifications, including ThyGly, 5,6-dihydroxy-5,6-dihydrouracil (UraGly), 5-hydroxycytosine (5-OHCyt), 5-hydroxy-5-methylhydantoin (5-OH-5-MeHyd) and 5-hydroxyhydantoin (5-OHHyd) (D’Ham et al., 1999; Cadet et al., 2000; Gasparutto et al., 2009). In contrast to HPLC based methods, the latter assays with DNA glycosylases minimize the degree of artefactual oxidation because it greatly reduces the release of potential oxidants attached to DNA and nonmodified monomers that may be subject to oxidation, i.e., DNA remains mostly intact. Furthermore, a second major advantage of these biochemical tools is their low background and high sensitivity that permits the accurate detection of very low steady-state levels of SSBs/oxidized bases as well as small variations in their frequency. This advantage was illustrated by the observed accumulation with age of modified guanine sites in the DNA liver of null *OGG1* mice using the modified alkaline elution technique, information that was not accessible by HPLC-ECD measurements (Klungland et al., 1999). Another relevant example concerned the observation of a biphasic curve for the decrease with time of the frequency of OGG1-sensitive sites by applying the enzymatic version of the alkaline elution method (Osterod et al., 2001). In contrast, attempts to assess the repair kinetics have failed using HPLC-ECD because of the inability to measure physiologically relevant levels.

Numerous applications of the modified enzymatic methods with emphasis on the alkaline comet assay have been devoted to model studies with single cells as a means to monitoring changes in the steady-state levels of oxidatively generated damage to DNA associated with environmental factors/health issues on large human cohorts (Møller and Roursgaard, 2021). However, quantitative measurement of the damage frequency requires the calibration of enzymatic assays (Møller et al., 2018). This is usually achieved by assuming that 1 Gy of low LET ionizing radiation generates 0.31 DNA strand breaks/alkali-labile sites per 10^9^ Dalton of mammalian genomes and corresponds to 1,000 breaks per diploid cell (Ahnstrom and Erixon, 1981). Another possibility is to establish a positive control using either Ro19-8,022 a quantitative ^1^O_2_ photosensitizer, or potassium bromate, a specific one-electron oxidant of guanine (Murata et al., 2001). The steady state values obtained by the modified elution technique (0.6–2.0 lesions/10^6^ dG) as well as observed increases of lesions with age in rodent cells and tissues are comparable to those obtained by HPLC-ECD using the NaI method of extraction (Helbock et al., 1998; Hamilton et al., 2001). Thus, it is not clear whether the inclusion of NaI in the HPLC-ECD and LC-MS/MS methods minimizes artificial oxidation to the level that can be useful for physiological studies. From studies using enzymatic methods though, the large reduction in artifactual oxidation renders the assays more suitable for studies of DNA damage induced by ionizing radiation and genotoxic compounds, and associated studies investigating the activity of DNA repair enzymes.

#### 3.1.3 Sequencing methods for mapping oxidized bases at the single base resolution

Epigenetic 5-HmdCyt, the TET oxidation product of 5-MeCyt that is preferentially formed at CpG sites in the genome is considered as the sixth most abundant DNA nucleobase (Song and He, 2011). Stable 5-HmCyt is prevalently generated in embryonic stem cells (ESC) and brain tissue at a frequency close to 10% of 5-mCyt sites. Rapidly after the discovery of this major oxidized nucleobase that is refractory to repair through the BER pathway, attempts were made to map at the single nucleoside resolution 5-HmCyt and its 5-mCyt precursor in biologically relevant gene sequences (Jin et al., 2010; Pastor et al., 2011; Song et al., 2011; Wu et al., 2011; Xu et al., 2011). The dynamic distribution of these two major epigenetic marks remains a challenging analytical issue and has been the subject of numerous investigations during the last decade (for recent reviews see Erlitzki and Kohli, 2024; Song et al., 2025). For this purpose, three main strategies have been considered. The widely used method of bisulfite sequencing (BS-seq) does not allow the distinction between 5-mCyt and 5-HmCyt (Jin et al., 2010). This limitation was overcome by the development of TET-assisted bisulfite sequencing (TAB-seq) (Yu et al., 2012) and oxidative bisulfite sequencing (oxBS) techniques (Booth et al., 2013). Other improvements have been made by optimizing the chemistry and including enrichment of methylated regions of the genome (Ficz et al., 2011; Pastor et al., 2011; Robertson et al., 2011; Song et al., 2011; Stroud et al., 2011; Williams et al., 2011; Wu et al., 2011). There is presently an array of methods that integrate the analysis of 5-mCyt and 5-HmCyt at base resolution into third-generation sequencing platforms, which include single molecule real-time (SMRT) sequencing (Ardui et al., 2018; Hu et al., 2025), nanopore sequencing (Wang et al., 2021) and chemical modification coupled with isothermal CRISPR-based assay (Zhang et al., 2022; Zou et al., 2025). Meanwhile, sequencing at the base resolution of the other oxidation products of TET, 5-FoCyt and 5-CaCyt, remains highly challenging since they are generated with a low abundance of about 1 lesions per 10^−6^ Cyt. This remark applies as well to available methods for mapping the distribution of 8-oxoGua at single base resolution (Riedl et al., 2016; Zhang et al., 2022; Dong et al., 2022; Dong et al., 2025; Ji et al., 2025).

### 3.2 Oxidatively generated damage in cellular DNA

As previously mentioned, the measurement of oxidized bases/2′-deoxyribonucleosides in cellular DNA that are formed in very low amounts remains a challenging analytical issue (Collins et al., 1997). The use of inappropriate methods including GC-MS, immunoassays and post-labeling methods has led to false conclusions for both qualitative and quantitative aspects of the formation of oxidatively generated base damage in cells and tissues. This is the case of 2-hydroxyadenine, 5,6-dihydroxycytosine, 5-hydroxy-5,6-dihydrothymine and 5-hydroxy-5,6-dihydroxyuracil whose formation was initially substantiated based on questionable GC-MS measurements (Dizdaroglu, 2012; Halliwell et al., 2021). The formation of the latter products has not been confirmed by LC-MS/MS analysis of cellular DNA exposed to oxidizing agents including ionizing radiation. Another source of artefactual contribution that cannot be totally prevented is the adventitious oxidation of overwhelming canonical nucleobases during DNA extraction and the subsequent enzymatic digestion steps preceding chemical analyses. To assess artefactual oxidation, a recent study reported dividing the DNA sample into two fractions, such that, one fraction is treated with Fpg before complete enzymatic digestion and thereby serves as a control for the other non-treated fraction (Dong et al., 2025). Nevertheless, artefactual oxidation can affect the suitability of chromatographic methods for monitoring oxidatively induced base modifications and explains why only a few accurate measurements have been performed so far as reported in the next section. To achieve this goal, two main fulfilments should be observed, which include evidence showing the effect of acute conditions of oxidative stress and establishment of dose/response curves.

#### 3.2.1 Radiation-induced base modifications

Ionizing radiation is a suitable tool to trigger, in a highly quantitative and well-controlled way, the formation of DNA oxidation modifications through the generation of reactive ·OH and ionization of the bases/2-deoxyribose moieties. However, multiple radical/excitation events are generated by the impact of high energetic photons along the radiation track (O’Neill and Wardman, 2009; Georgakilas et al., 2013). These indirect and direct processes result in the formation of complex DNA damage that include in addition to deleterious double strand breaks (DSB) non-DSB oxidatively generated clustered damage consisting of base modifications, single strand breaks and oxidized abasic sites within one/two helix turns (Cadet et al., 2012; Sage and Shikazono, 2017). The latter lesions are expected to be unique when considering the sequence context and the fact that more than 15 oxidized bases have been identified so far, thus preventing the identification of non-DSB oxidatively generated DNA damage. Noteworthy, nuclease mediated digestion of oxidized DNA suppresses the complexity of the clustered modifications and leads to the release of modified 2′-deoxyribonucleosides/nucleobases that are accurately characterized by LC-MS/MS analysis (Cadet et al., 2017).

**FIGURE 1:**
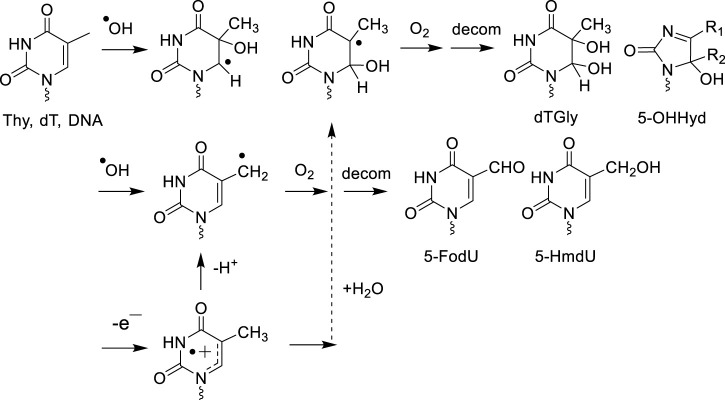
^**.**^OH and one-electron oxidant decomposition pathway of thymine.

**FIGURE 2:**
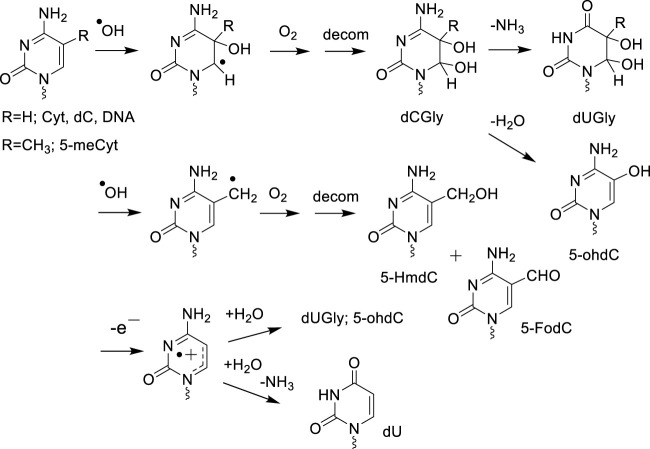
^**.**^OH and one-electron degradation pathway of cytosine and 5-methylcytosine.

Mechanistic studies on DNA model systems in aqueous solutions have shown a qualitative similarity in the distribution of the nucleobase oxidation products formed by ·OH and one-electron oxidants (Cadet and Wagner, 2014). For example, the addition of ·OH at the 5,6-double bond of pyrimidines (Thy, Cyt and 5-mCyt) as well as the hydration of pyrimidine radical cations gives rise to analogous hydroxylated radical adducts that subsequently transform into the corresponding 5,6-hydroxyperoxyl radical intermediates in the presence of O_2_ (Figures 1, 2). The stable final degradation products of 5,6-hydroxyperoxyl radical intermediates mostly include the four *cis* and *trans* diastereomeric dTGly together with 5*R*- and 5*S* 1-(2-deoxy-β-D-*erythro-*pentofuranosyl)-5-hydroxyl-5-methylhydantoin (5-OH-5-mCHyd) that are generated as the stable final degradation products of Thy (Frelon et al., 2000; Cadet et al., 2017). The situation is more complex for cytosine and 5-methylcytosine oxidation products since in addition to 5,6-dihydroxy-5,6-dihydro-2′-deoxyuridine (dUGly), the deamination product of unstable 5,6-dihydroxy-5,6-dihydro-2′-deoxycytidine (dCGly) (Tremblay et al., 1999), the four *cis* and *trans* diastereomeric 2′-deoxyribonucleosides of N1-carbamoyl-2-oxo-4,5-dihydroxyimidazolidine and N1-carbamoyl-2-oxo-4,5-dihydroxy-5-methylimidazolidine (Samson-Thibault et al., 2012; Madugundu et al., 2014) are formed through rearrangement of unstable 5-hydroxy-6-hydroperoxy-5,6-dihydro-2′-deoxycytidine and 5-hydroxy-6-hydroperoxy-5,6-dihydro-5-methyl-2′-deoxycytidine, respectively (Tremblay et al., 2007; Wagner and Cadet, 2010). As an additional oxidizing degradation pathway, competitive deprotonation of the methyl group of dT and 5-mdC radical cations was found to generate 5-HmdU/5-fodU and 5-HmdC/5-fomdC through the intermediacy of 5-(uracilyl) methyl and 5-(cytosilyl) methyl radical intermediates, respectively (Figure 2).

Several stable 2'-deoxyribonucleoside modifications were accurately detected by LC-MS/MS in the DNA of γ-ray exposed Fischer glioma cells (F98) and human monocytes with a linear dose dependence for elevated radiation doses ranging from 100 Gy to 3 kGy. The formation yields expressed in the number of modifications per 10^9^ normal nucleosides are reported in Table 1.

**TABLE 1:** Formation of oxidatively generated base damage in cellular DNA by ionizing radiation and high intensity UVC nanosecond laser pulses: number of modifications per 10^9^ nucleosides per Gy or per pulse.

	Gamma rays^a^	Gamma rays^b^	UVC laser^c^
dTGly	97	7.7	160
5-HmdU	29	13.3	60
5-FodU	22	19.5	20
8-oxodAdo	3	d	d
FapyAde	5	d	d
8-oxodGuo	20	8.8	1,290
FapyGua	39	d	d
5-HmdC	d	1.2	d
5-FodC	d	0.7	d

^a^ from Pouget et al., 2002.

^b^ from Madugundu et al., 2014.

^c^ from Douki et al., 2004.

^d^ not determined.

**FIGURE 3:**
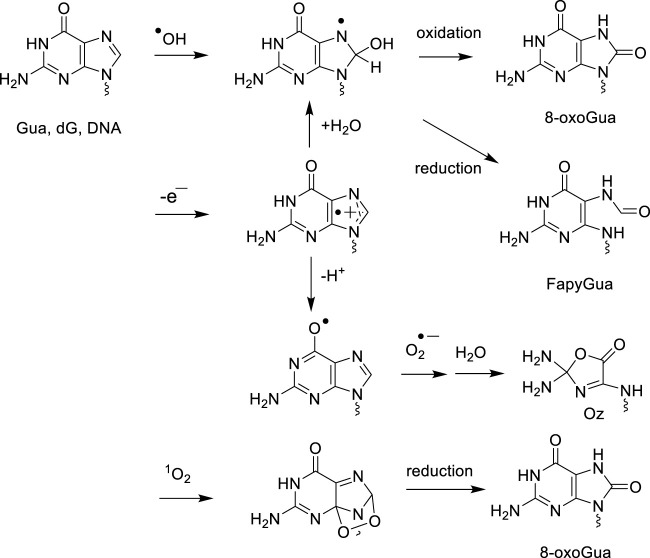
Oxidatively generated decomposition pathways of guanine by ^**.**^OH, one-electron oxidants and singlet oxygen.

Like pyrimidines, there is also a high similarity between the ·OH and one-electron oxidation mediated degradation pathways of adenine and guanine in both isolated and cellular DNA (Cadet and Wagner, 2014). Hydration of purine base radical cations that is favored in double stranded DNA led to the formation of reducing 8-hydroxy-7,8-dihydropurinyl radicals, which are also generated by the addition of ·OH to C8 of Gua and Ade. One-electron oxidation of the latter intermediates gives rise to 8-oxoGua and 8-oxo-7,8-dihydroadenine (8-oxoAde) while competitive reduction of 8-hydroxy-7,8-dihydropurinyl radicals that is enhanced in cellular DNA, produces 2,6-diamino-4-hydroxy-5-formamidopyrimidine (FapyGua) and 4,6-diamino-5-formamidopyrimidine (FapyAde) through opening of the imidazole ring (Cadet et al., 2017) (Figure 3). The guanine degradation products are formed in cellular DNA in about 10-fold higher yields than the corresponding adenine lesions as observed in isolated DNA and double stranded oligonucleotides. This could be partly explained by the addition of pyrimidine peroxyl radicals to C8 of vicinal guanine, reactions that lead after rearrangement to the formation of 8-oxoGua and FapyGua containing tandem base lesions (Robert et al., 2023). Deprotonation of the guanine radical cation leads to transient and highly oxidizing Gua (-H)· radicals that upon addition of O_2_·- to C5 gives rises to 2,2,4-triamino-5(2*H*)-oxazolone (Oz) through a complex sequence of reactions (Misiaszek et al., 2004; Cadet et al., 2008). Formation of related 2'-deoxyribonucleoside that was monitored in the DNA of diabetic rats (Matter et al., 2006) has not been yet measured in gamma-irradiated cells. From a quantitative distribution of the main radiation-induced base decomposition products with that of one-electron oxidation (see next section), it was concluded that indirect effects of gamma rays predominate over ionization mediated by direct interactions of energetic photons with DNA (Pouget et al., 2002; Douki et al., 2006; Cadet et al., 2022). This was further supported by the observed decrease in the yield of 8-oxodG and dTGly with an increase in the linear energy transfer (LET) of ^12^C^6+^ (24.5 keV/μm) and ^36^Ar^18+^ (250 keV/μm) heavy charged particles in comparison to gamma rays, i.e., high LET radiation triggers a concomitant lowering in the yield of ·OH. Other radiation-induced damage identified in the DNA of human lymphocytes consists of the four *cis* and *trans* diastereomeric aldehyde adducts to cytosine (Regulus et al., 2007) that were initially detected in ^**.**^OH-mediated oxidation of isolated DNA by LC-MS/MS operating in neutral scan mode (Regulus et al., 2004). Assignment of previously unknown enzymatically released 2′-deoxyribonucleosides to 6-(2-deoxy-β-D-*erythro*-pentofuranosyl)-2-hydroxy-3 (3-hydroxy-2-oxopropyl)-2,6-dihydroimidazo [1,2-c]-pyrimidin-5(3H)-one was achieved through extensive NMR analyses and exact high resolution mass spectrometry (HRMS) on line with HPLC (Regulus et al., 2007). As a relevant mechanistic insight, radiomimetic bleomycin that mostly acts by abstracting a hydrogen atom at C4′ of the 2-deoxyribose moiety (Dedon and Goldberg, 1992) was also shown to trigger the formation of these modifications. It was proposed that the resulting C4′-oxidized abasic site (Chen et al., 2007) or more likely its ring opened form (Pogozelski and Tullius, 1998) is able, when present in front of opposite cytosine or adenine, to be converted into a conjugated keto-aldehyde following beta elimination and the formation of a DNA strand scission on the 3′-end (Sczepanski et al., 2008). This results in the formation of cytosine cycloadducts as a function of either radiation dose or bleomycin concentration as part of complex deleterious lesions consisting of an intra-strand cross-link and a vicinal single strand break. The formation efficiency of dC aldehyde adducts within the 75–300 Gy dose range was one hundred-fold lower than that of 8-oxodG (Regulus et al., 2007).

#### 3.2.2 Questionable radiation-induced formation of 5′,8-purine cyclo-2′-deoxyribonucleosides in cellular DNA

The 5*R* and 5*S* diasteromers of 5′,8-cyclo-2′-deoxyadenosine (cdA) and 5′,8-cyclo-2′-deoxyguanosine (cdG) that arise from intramolecular cyclization of ·OH-mediated deoxyribos-5-yl radical to C8 of adenine or guanine nucleosides/nucleotides were identified as minor degradation products of calf thymus DNA in aerated aqueous solution (Belmadoui et al., 2010; Krokidis et al., 2017). Major interest has been devoted to the putative biological relevance of cdA and cdG during the past 2 decades following the report that cdG and cdA are repaired in model studies by the nucleotide repair excision (NER) pathway and not by BER operating on single oxidized bases (Brooks et al., 2000; Kuraoka et al., 2000). Several attempts to measure cdA and cdG in the DNA of mammalian cells by GC-MS, LC-MS and LC-MS/MS ELISA immunodetection have led to inconsistent conclusions with estimated levels that vary by two orders of magnitude (for a review, see Cadet et al., 2019a). Other questionable data that were estimated by LC-MS/MS concern the radiation-induced formation of 5′*R* and 5′*S* diastereomers of cdA and cdG in the DNA of MCF-7 and MDA-MB-231 breast cells in which differences were observed even upon exposure to 5 Gy. These results sharply contrast with the lack of detection of cdA and cdG in gamma irradiated human monocytes (Belmadoui et al., 2010) and *Thermococcus gammatolerans* (Barbier et al., 2016) at doses of 2 kGy and 5 kGy, respectively. A likely explanation for such major divergences is that the claimed formation of cyclic purine lesions in breast cells (Krokidis et al., 2017; Chatgilialoglu, 2019) resulted from spurious oxidative reactions associated with DNA extraction and its subsequent digestion before LC-MS/MS analysis (Cadet et al., 2019a; Cadet et al., 2019b). This clearly shows that formation/dose response information is required to further ascertain the formation of minor oxidatively generated damage to cellular DNA. Other confusing data deal with the measurement of cdA and cdG using ELISA in brain, liver and kidney tissue of healthy young rats. The reported values of formation between 2 and 5 lesions per 10^6^ nucleosides (Mori et al., 2019) are several-fold higher than the yield of 8-oxodG. This is not compatible with the expected 8-oxodG/cdA and 8-oxodG/cdG ratios of about 100 (Belmadoui et al., 2010).

#### 3.2.3 One-electron oxidized base modifications

As already mentioned, the direct interaction of gamma rays and heavy charged ions with DNA is able to ionize purine and pyrimidine nucleobases as well as the 2-deoxyribose moieties of DNA (Cadet and Wagner, 2013). Other biologically relevant one-electron oxidants that essentially act on nucleobase with a preference in most cases for guanine include type I photosensitizers (Baptista et al., 2017; Baptista et al., 2021), two-quantum UVC laser photolysis (Nikogosyan, 1990), metabolized bromate (Murata et al., 2001; Kawanishi and Murata, 2006; Ballmaier and Epe, 2006) and carbonate radical anions (Lee et al., 2007; Radi, 2013; Roginskaya et al., 2015; Matter et al., 2018), the decomposition product of nitrosoperoxycarbonate arising from the reaction of CO_2_/bicarbonate with peroxynitrite (Uppu et al., 1996; Illes et al., 2019).

Only a few examples of specific reactions of one-electron oxidants on cellular DNA are available. Visible light excited riboflavin, a preferential type I photosensitizer, has been shown by HPLC-ECD to induce the formation of 8-oxodG in the DNA of mammalian cells with a photosensitizer concentration and duration exposure dependence (Yamamoto et al., 1992; Bessho et al., 1993). Hydration of the initially generated Gua radical cation is the key step leading to 8-oxoGua as shown by mechanistic studies on isolated DNA and double stranded oligonucleotides (Kasai et al., 1992; Rokhlenko et al., 2014). FapyGua and 2,2,4-triamino-5(2*H*)-oxazolone, two other expected major decomposition products from the one-electron oxidation of Gua (Douki and Cadet, 1999) have not yet been measured in cellular DNA upon type I photosensitized oxidation. A suitable quantitative way to generate purine and pyrimidine base radical cations in both isolated and mammalian cells is provided by high intensity nanosecond 266 nm laser irradiation. Extensive photophysical studies on isolated 2′-deoxyribonucleosides has shown that under these conditions the purine and pyrimidine radical cations are generated with a similar efficiency (Angelov et al., 1997; Douki et al., 2001; Spassky and Angelov, 2002). This is rationalized in terms of further absorption of a UVC photon by the long-lived triplet excited nucleobases following intersystem crossing of initially single excited transients. Thus, the elevated level of absorbed energy that is higher by more than 2 eV above the ionization threshold of purine and pyrimidine bases leads to their efficient one-electron oxidation according to a bi-photonic process (Douki et al., 2004; Angelov et al., 2005). High intensity UVC laser irradiation of native CT DNA was shown to give 8-oxodG as the predominant oxidation product over minor products: dTGly, 5-HmdU, 5-FodU and 8-oxodA. However, pre-heated treatment of DNA before laser exposure leads to a total loss in the specific damage distribution since dTGly diastereomers were formed with a higher yield than 8-oxodG. The preferential formation of 8-oxodG in native DNA duplex is accounted for by a redistribution of initially generated base radical cations through positive hole migration with specific trapping of the hole by guanine bases that exhibit the lowest oxidation potential among DNA components. A similar oxidatively generated base damage distribution with an overwhelming formation of 8-oxodG was observed in the DNA of TPH1 human monocytes (Pouget et al., 2002; Douki et al., 2006) and HeLa cells (Madugundu et al., 2013) exposed to high intensity UVC ns laser pulses (Table 1). This strongly gives support to the notion that charge transfer occurs in cellular DNA upon one-electron oxidation of the nucleobases as previously shown in isolated DNA duplexes (Genereux and Barton, 2010; Kanvah et al., 2010). In addition, the formation of intra-strand cross-links generated by covalent addition of non-adjacent thymine at N3 to Gua·+/G (-H)^**.**^ at C8 was observed by LC-MS/MS measurement, albeit at low levels of less than 1% of 8-oxodG.

The characteristic distribution profile of modified nucleobases in one-electron oxidized cellular DNA shows the predominance of 8-oxodG and likely FapyGua whose formation however remains to be assessed (Madugundu et al., 2013). A comparison of the distribution of base modifications generated by one-electron oxidation and the direct/indirect effects of gamma rays in cellular DNA supports the proposal that the ionization of bases contributes to a lower extent than ·OH to the formation of radiation-induced damage (Douki et al., 2006; Cadet et al., 2019c). A preference toward guanine damage in DNA may also arise from the reaction of ^**.**^OH with bicarbonate present in cells, thereby, diverting the oxidation of DNA from that with ^**.**^OH to that with one-electron oxidizing carbonate radical (Fleming and Burrows, 2022). On the basis of competition kinetics, however, it is not likely that bicarbonate can sufficiently scavenge ^**.**^OH to make a significant difference (Halliwell et al., 2021). The situation is different with Fenton-induced oxidation of DNA because bicarbonate strongly interacts with Fe^2+^ and gives carbonate radical anions upon reaction with H_2_O_2_ as inferred by studies in cells exposed to H_2_O_2_ (Fleming et al., 2024). In support of a major contribution of radiation-induced ^**.**^OH, the levels of both Fpg and Endo III-sensitive as contributors to the steady-state DNA damage were measured in similar yields in control THP1 cells using the modified alkaline comet assay (Pouget et al., 1999).

#### 3.2.4 Singlet oxygen guanine oxidation modifications

^1^O_2_ in its first excited state, singlet oxygen (^1^Δg) reacts specifically with guanine to generate overwhelmingly 8-oxoGua (Figure 3) in isolated DNA (Ravanat et al., 2001; Dumont et al., 2016; Di Mascio et al., 2019) at the exclusion of dSp that is predominantly produced with single Gua monomeric components and oligonucleotides (Cadet et al., 2008). Similarly, using thermolabile naphthalene endoperoxide as a source of |^18^O]-labeled ^1^O_2_, it was shown that only 8-oxodG is formed upon incubation of TPH1 monocytes with the oxidizing agent (Ravanat et al., 2000). Numerous examples of the efficient formation of 8-oxoGua upon exposure to UVA excited photosensitizers in mammalian cells have been reported on the basis of HPLC-ECD and LC-MS/MS measurements (for extensive reviews see Cadet et al., 2005; Di Mascio et al., 2019; Baptista et al., 2021). UVA radiation and to a lesser extent blue light have been shown to take place through a major contribution of type II photosensitization, giving ^1^O_2_ that subsequently produces 8-oxodG in both cellular DNA and skin explants (Douki et al., 1999; Douki et al., 2003; Mouret et al., 2006; Cadet et al., 2024).

#### 3.2.5 TET-mediated oxidation modifications of 5-methylcytosine

Epigenetic 5-HmdC is the initially formed TET-mediated oxidation product of 5-medC as part of the active demethylation process within DNA (Tahiliani et al., 2009). The level of 5-HmdC by at least 2 to 3 orders of magnitude higher than other chemically and enzymatically oxidized 2′-deoxyribonucleosides underscores this modification as a stable epigenetic biomarker. Because of its high levels, 5-HmdC has been accurately measured in the DNA of animal issues by 1D- and 2D- LC-MS/MS (Pfaffeneder et al., 2014; Gackowski et al., 2015; Liu et al., 2016) and mapped in gene sequences at the nucleoside level (Kisil et al., 2024). Attempts were made to evaluate this biomarker as a potential indicator of cancer and aging (Kraus et al., 2012; Wagner et al., 2015; Song et al., 2022). Interestingly, the level of 5-HmdC in DNA is also modulated by vitamin C, which is a co-factor in the TET-mediated oxidation of 5-medC (Young et al., 2015; Guz et al., 2024). In contrast to 5-HmdC, the further oxidation products of TET-mediated oxidation of 5-mdC, which include 5-FodC and 5-CadC, are present at low steady state levels of about a few lesions per 10^6^ nucleosides. Because of the low levels of these modifications, their analysis by LC-MS/MS remains to be challenging. On the other hand, it remains to be established whether 5-HmdU that has been proposed to be generated with a low efficiency by TET oxidation of thymine in DNA (Pfaffeneder et al., 2014) does not arise mostly from artefactual contribution prior to analysis.

## 4 Perspectives

Major progress has been made during the two last decades toward the identification and analysis of modified bases/nucleosides formed in DNA upon exposure to ^**.**^OH, one-electron oxidants and ^1^O_2_. This has largely been achieved by LC-MS/MS measurements, which can assess the formation of several modifications and provide relevant mechanistic insights into the molecular effects of UVA/visible photons, high intensity UVC laser pulses and ionizing radiation. The similarity in the distribution of oxidatively-induced base damage in cellular and isolated DNA (Cadet et al., 2017) allows one to conclude that these model studies are appropriate for describing the mode of action of chemical and TET enzymatic oxidants in cellular DNA. However, strong oxidizing conditions through acute exposure to chemical and physical agents are necessary to accurately obtain a comprehensive profile of persistent modifications in cellular DNA. Application of these rigorous approaches have questioned the formation and therefore the biological relevance of several oxidized nucleosides including 2-hydroxy-2′-deoxyadenosine, 5′,8-cyclo-2′-deoxyadenosine and 5′,8-cyclo-2′-deoxyguanosine. It may be added that the enzymatic modified comet assay and alkaline elution technique provide complementary information on the steady-state level of oxidatively generated DNA damage resulting from oxidative metabolism and mild conditions of chronic exposure to oxidants. In general, however, LC-MS/MS and biochemical based assays remain to assess the formation in cells of several classes of oxidatively generated damage that have been identified in DNA model studies. These include 8-oxodG as an ozone-mediated product oxidation (Wagner et al., 2021), tandem lesions arising from the addition of pyrimidine peroxyl radical to vicinal intra-strand guanine (Robert et al., 2023) and one-electron induced generation of DNA-protein cross-links through the covalent addition of lysine to guanine radical cation (Perrier et al., 2006). A better assessment of the structure and quantities of complex lesions that are produced by ionizing radiation and other genotoxic agents in chemo- and radiotherapy may help to improve the design of treatment protocols in medicine. An interesting challenge today is to understand the modifying effects on the degradation pathways of cellular DNA at elevated dose rates of ionizing radiation associated with ‘FLASH’ radiotherapy.
